# Bioequivalence assessment between two formulations of a film-coated fixed-dose combination of metformin and vildagliptin (850/50mg) in healthy Tunisian subjects under fed conditions

**DOI:** 10.1038/s41598-025-34082-4

**Published:** 2026-02-06

**Authors:** Khouloud Ferchichi, Emna Gaies, Syrine Ben Hammamia, Hanene El Jebari, Mouna Daldoul, Salma Ghorbel, Semia Srairi, Sami Guermazi, Yousr Gorgi, Ilhem Boutiba, Kahena Bouzid, Aimen Abbessi, Riadh Daghfous, Mouna Ben Sassi, Sameh Trabelsi

**Affiliations:** 1 Department of Clinical Pharmacology, National Center Chalbi Belkahia of Pharmacovigilance, Rue Zouhaier Essafi, 1006 Tunis, Tunisia; 2 Research Laboratory of Clinical and Experimental Pharmacology (LR16SP02), Tunis, Tunisia; 3 Faculty of Medicine of Tunis, University of Tunis El Manar, 1007 Tunis, Tunisia; 4 Hematology Medical Laboratory at Charles Nicolle Hospital, Tunis, Tunisia; 5 Immunology Medical Laboratory at Charles Nicolle Hospital, Tunis, Tunisia; 6 Microbiology Medical Laboratory at Charles Nicolle Hospital, Tunis, Tunisia; 7 Biochemistry Medical Laboratory at Charles Nicolle Hospital, Tunis, Tunisia; 8 Pharmacy at Charles Nicolle Hospital, Tunis, Tunisia

**Keywords:** Metformin, Vildagliptin, Bioequivalence, Tunisia, Bioavailability, Diabetes, Diseases, Drug discovery, Health care, Medical research

## Abstract

In Tunisia, bioequivalence studies have been required for generic drug approval since 2008. This study aimed to assess the bioequivalence of vildagliptin and metformin fixed-dose combination (FDC) 850/50 mg for a tested (Bi-Galvine) versus the approved reference products (Galvumet). A randomized, two-way, two-period, single oral dose, open-label, crossover, bioequivalence study with a washout period of 7 days to compare metformin/vildagliptin film-coated tablets in 18 healthy Tunisian subjects aged between 18 and 50 years under fed conditions was conducted at the National Center Chalbi Belkahia of Pharmacovigilance. During each period, 20 blood samples were collected from each subject at pre-dosing (0.00) and between 0.25 and 24.00 h after dosing. The bioequivalence between the test (T) and reference (R) products required 90% confidence intervals (CIs) for the geometric least square (LS) mean T/R ratio to be within 80–125% for the pharmacokinetic parameters, maximum plasma concentration (C_max_), and Area Under the Curve from zero to 24 h (AUC_0–24 h_). The 90% CIs of the geometric means of the T/R ratios for C_max_ and AUC_0–24 h_ for metformin were 92.01–102.66% and 93.55–101.80%, respectively; the corresponding results for vildagliptin were 96.03–107.09% and 94.46–101.92%, respectively. Other parameters, such as AUC_0-∞_, time to maximum concentration (T_max_), and terminal half-life (t_1/2_), were comparable between the test and reference products. Adverse events (AEs), mainly hypoglycemia and loose stools events, were reported without relevant differences between the test and reference products. AEs were generally mild and transient. No severe or serious AEs occurred. The new generic drug product of metformin/vildagliptin FDC 850/50 mg demonstrated bioequivalence to the approved product and is therefore expected to provide similar therapeutic effects.

## Introduction

Diabetes mellitus is a chronic disease that poses a major public health issue with a steadily rising prevalence, making it one of the top healthcare system concerns^1^. Clinical trials have shown that adjuvant vildagliptin treatment in patients with inadequate glycemic control despite metformin monotherapy induced statistically significant mean reductions in glycosylated hemoglobin (HbA1c) compared with placebo^2^. The development of a fixed-dose combination (FDC) of metformin and vildagliptin is a strategic approach to address the complex challenges of diabetes management, providing effective glycemic control while potentially improving patient adherence and reducing healthcare costs. Ensuring the availability of a safe and effective locally manufactured FDC is particularly important for the Tunisian population.

This clinical trial was therefore designed to evaluate the bioequivalence between the test product, Bi-Galvine 850/50 mg Film-Coated Tablets (SAIPH Pharma, Tunisia), and the reference product, Galvumet 850/50 mg Film-Coated Tablets (Novartis Pharma Schweiz AG, Risch, Switzerland). Demonstrating bioequivalence ensures therapeutic interchangeability and constitutes a regulatory requirement for marketing authorization of the test product according to Tunisian health authority guidelines. The primary objective of the study was to compare the rate and extent of absorption of metformin and vildagliptin between the test and reference formulations after a single oral dose under fed conditions.

## Materials & methods

The study was conducted at the Bioequivalence Unit of the Clinical Pharmacology Department affiliated with The National Center of Pharmacovigilance in Tunisia, according to the Declaration of Helsinki and Good Clinical Practice guidelines^3,4^. The study protocol was approved by the Committee of Persons Protection - Tunisia North (approval no. CPPN_15_2022_AMD_BiGalvineFed). The study was registered on ClinicalTrials.gov under the number: NCT06103747 on 17th December 2022 (https://clinicaltrials.gov/study/NCT06103747?cond=metformin&lat=36.8045507&lng=10.1654236&locStr=Bab%20Souika,%20Tunis,%20Tunisia&distance=50&rank=3#more-information).

### Study drugs

The test product Bi-Galvine 850/50 mg Film-Coated Tablets, was manufactured by SAIPH Pharma, Tunisia, batch number 1M235 and expiry date is March 2024.

The reference product was Galvumet 850/50 mg Film-Coated Tablet of the Marketing Authorization Holder: Novartis Pharma Schweiz AG, Risch, batch number KEV82 and expiry date is November 2023.

## Study design

This study was an open-label, randomized, single-dose, two-treatment, two-period, two-sequence, crossover bioequivalence study under fed conditions with a washout interval of one week between periods. For fixed-dose combinations containing metformin—such as metformin/vildagliptin—both the **FDA** and **EMA** guidelines^5^ specify that bioequivalence should be demonstrated under the conditions stated in the product labeling of the reference product. Galvumet is labeled to be taken with food to reduce gastrointestinal side effects of metformin^6^. Therefore, a fed study is required to match real clinical use and to ensure fair comparison of test and reference products.

In each study period, the drugs were administered according to the randomization plan with 240 ± 2 mL of warm 20% glucose solution. The subjects’ ingestion of the dose was checked and verified. The subjects stayed in bed in an upright position for the first 4 h after dosing.

During each study period, the subjects fasted for at least 10 h and then ate breakfast prior to drug intake, and food was not allowed for 6 h after drug administration. Standardized high-fat breakfast (800–1000 calories) was served 30 min before dosing. During their housing, the subjects were not allowed to have meals or fluids other than those served by the study team. All meals were identical in both periods and served at the same time.

## Study population

The study sample size was determined following recommendations from the bioequivalence guidelines of the FDA and the EMA^5,7^.

The sample size for this study was calculated considering the following data: within-subject variability driven from the literature data was 8.95% for metformin and 15.85% for vildagliptin^8^; the significance level was α = 5%; the desired statistical power (1−β) was 90% and the 90% confidence interval (90% CI) around the ratio of geometric means fall within the bounds of bioequivalence, typically [80.00–125.00%] for primary endpoint^5^.

The calculated sample size was 13^9^, accounting for potential dropouts or withdrawals we estimated a dropout rate of 25%. Thus, the final sample size was 18.

The study population consisted of healthy volunteers aged between 18 and 50 years.

Each subject was well informed about the study objectives, procedures, possible risks, and interests before providing written informed consent. Healthy subjects aged 18–50 years with body mass indices ranging from 18.5 to 30.0 kg/m^2^ were eligible to participate in the study. The health status of the subjects was assessed on the basis of vital signs (temperature, blood pressure, and pulse), medical history, routine physical examination, 12-lead electrocardiogram (ECG), clinical laboratory tests (hematology, blood biochemistry, coagulation function, urinalysis, drug screening), and serology (hepatitis B surface antigen, hepatitis C virus antibody, and HIV antigen/antibody). Subjects with any allergy, hypersensitivity history, known lactose intolerance, drug or alcohol abuse, or taking medications or food supplements within 2 weeks before the first dosing were excluded.

## Sample collection and analysis

During each period, 20 blood samples were collected from each subject at pre dosing (0.00) and at 0.25, 0.5, 0.75, 1, 1.5, 2, 2.5, 3, 3.5, 4, 4.5, 5, 6, 7, 8, 10, 12, 14 and 24 h after dosing under fed conditions. The samples were subsequently centrifuged at 4000 rpm for 5 min at 4 °C within 30 min after collection. The plasma tubes were then stored in an Ultra freezer (− 80 °C) until analysis. The plasma concentrations of both metformin and vildagliptin were measured via in-house previously validated liquid chromatography tandem mass spectrometry (LC‒MS/MS) according to ICH M10^10^. Protein precipitation with acetonitrile containing diazepam as an internal standard was used for plasma extraction of both metformin and vildagliptin.

Sample analysis was performed via LC‒MS/MS via an ODS HypersylTM 125*4.6 mm column and a Waters Acquity H CLASSP ULC® liquid chromatography pump with the following mobile phases: A, 300 mL ammonium formate + 300 µL formic acid; and B, 700 mL methanol + 700 µL formic acid at a flow rate of 0.8 mL/min.

Detection was performed via a Waters Triple Quad (TQ-S micro Xévo) detector. Mass spectrometry was performed with multiple reaction monitoring (M/z 130.128/60.04 for metformin, M/z 304.294/154.189 for vildagliptin and M/z 285.157/154.114 for diazepam). The linearity range of the method was 20–3000 ng/mL for metformin and 5–320 ng/mL for vildagliptin.

The LC–MS/MS methods used for measuring metformin and vildagliptin plasma concentrations were fully validated according to ICH M10 guidelines^10^, ensuring accuracy, precision, selectivity, linearity, and stability.

## Safety assessment

Safety and tolerability assessments included vital signs (temperature, blood pressure, and heart rate), physical examination, ECG, blood glucose, and laboratory tests at regular intervals throughout the study. Vital signs were measured before dosing at -12.00 and − 1.00 h and at 2, 4, 6, 8, 12, and 24 h after dosing. Blood glucose was measured at 1.5 h and 3.5 h after dosing. Physical examination, ECG, and laboratory tests (hematology, blood chemistry, and urinalysis) were performed at screening and at the end of the study.

Subjects were closely monitored for any adverse events (AEs) that may have occurred during the study from the initiation of any study procedures to the end of the study.

### Pharmacokinetic and statistical analyses

The primary study endpoints were maximum plasma concentration (C_max_), Area Under the Curve from zero to 24 h (AUC_0–24 h_) and from zero to infinity (AUC_0 – ∞_).

Non-compartmental analysis approach implemented in R (version 4.3.1) with the PKNCA^11^ package (https://cran.r-project.org/package=PKNCA) was performed to calculate these pharmacokinetic (PK) parameters as follows:

- The values of C_max_ and time to maximum concentration (T_max_) were obtained directly from the concentration versus time curve of each subject.
- The terminal elimination rate constant (K_e_) was estimated for each subject and for each drug substance via linear regression of the last points (at least three points were used) at the terminal phase of the log-concentration versus time curve of each subject.
- The terminal half-life (T_1/2_) was calculated from 0.693/K_e_.
- AUC_0 − 24 h_ was calculated via the the linear-up trapezoidal method.
- The extrapolated area, (AUC_24h−∞_) is the area under the plasma concentration‒time curve from 24 h to infinity and was calculated as the last concentration (C_last_) divided by the elimination rate constant C_last_/K_e_. It is also called the residual area expressed as a percentage of the AUC_0−∞_.
- AUC_0−∞_ was calculated from the sum of AUC_0 − t_ and AUC_t−∞_.

The assessment of bioequivalence was based upon 90% confidence intervals for the ratio of the population geometric means (test/reference) for the parameters under consideration. This confidence interval was then back-transformed to obtain the desired confidence interval for the ratio on the original scale.

For both metformin and vildagliptin, the 90% confidence intervals for C_max_ and AUC_₀–24 h_ lie within the acceptance range of 80.00–125.00%.

Analysis of variance (ANOVA) was performed to assess the effects of the following factors: period, sequence, subjects nested within sequence and treatment (formulation or product).

ANOVA was applied for the Ln-transformed values of C_max_, AUC_0–24 h_ and AUC_0−∞_ for metformin and vildagliptin. For each mentioned factor, the differences were not statistically significant at the 5% significance level (*p* ≥ 0.05).

The Residual Mean Square (MSE), which is the residual within-subject variance from the ANOVA on ln-transformed data, was used to estimate the intra-subject coefficient of variation (CV%) according to the regulatory guidelines^5^. The CV% was calculated separately for each pharmacokinetic parameter and reported as percentage.

## Results

### Demographic characteristics

Eighteen healthy subjects, 13 males and five females, were enrolled in this study as planned. The mean age was 28.61 ± 10.33 years. The mean BMI was 24.14 ± 3.22 kg/m. All the subjects were nonsmokers and had no concurrent alcohol consumption. All 18 subjects were randomized under fed conditions and completed both periods. All 18 subjects ware included in the pharmacokinetics analysis.

### Pharmacokinetic analysis

**Fig. 1:**
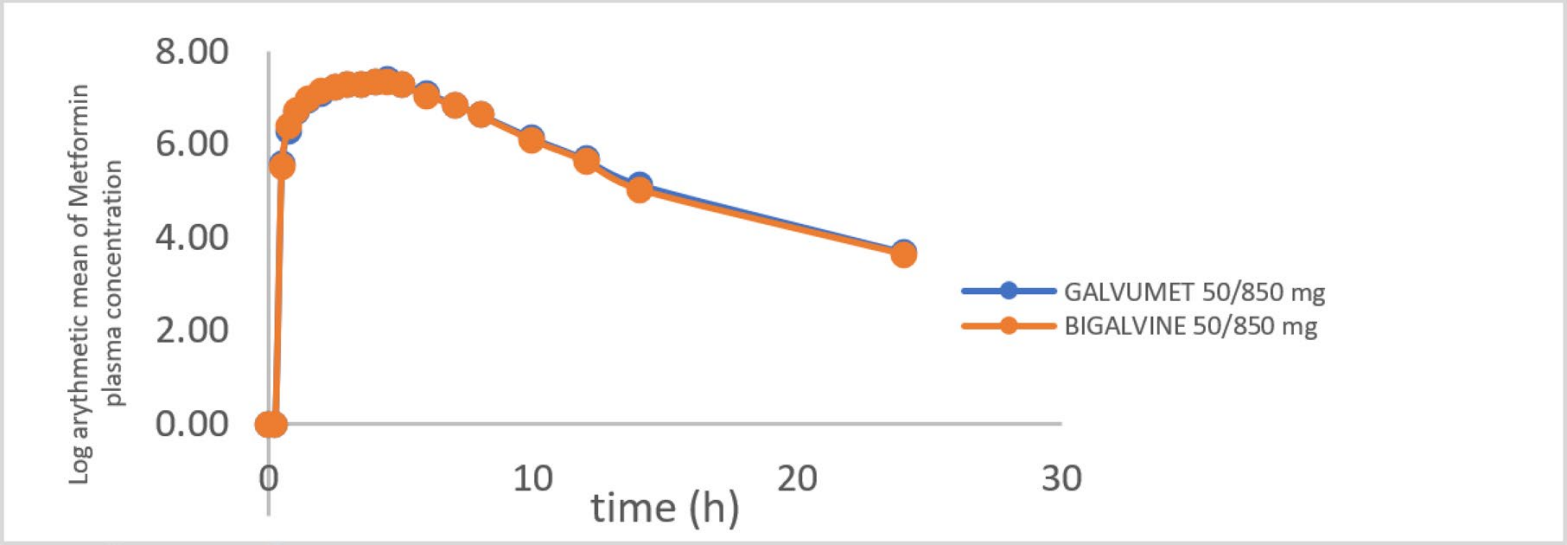
Logarithmic arithmetic mean plasma metformin concentration (ng/ml) versus time (hour) curve following a single oral dose of metformin and vildagliptin 850/50 mg Film-Coated Tablets.

**Fig. 2:**
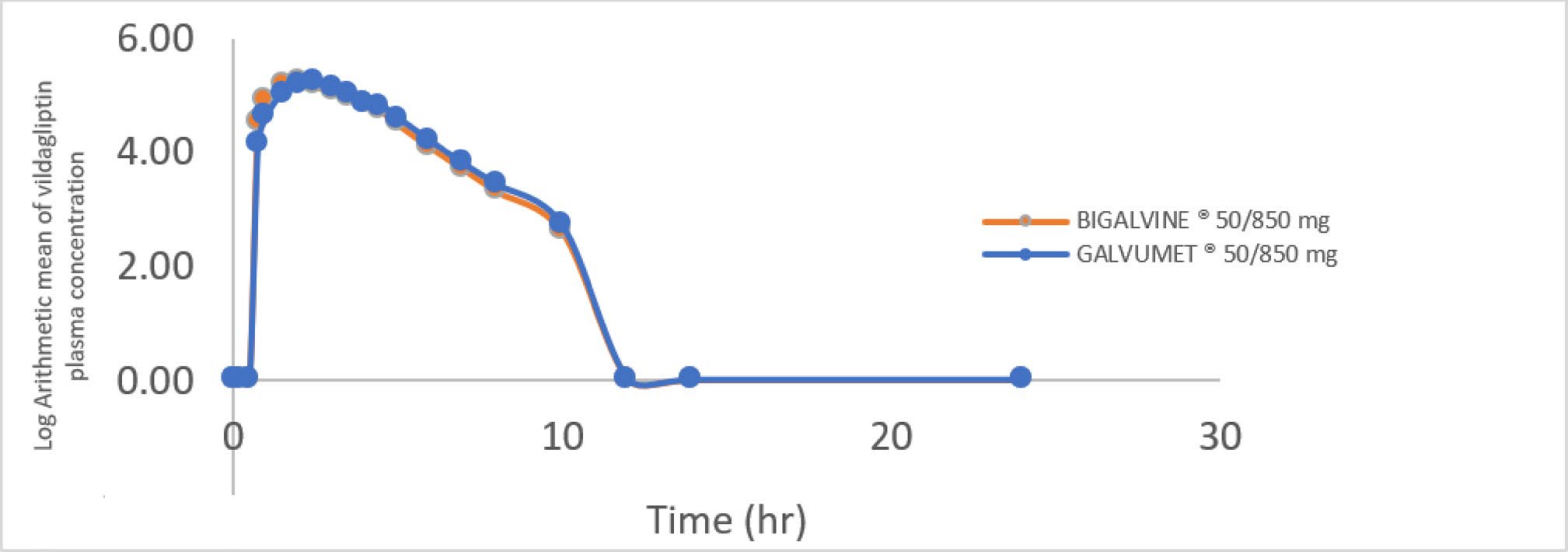
Logarithmic arithmetic mean of the plasma vildagliptin concentration (ng/ml) versus time (hour) curve following a single oral dose of metformin or vildagliptin 850/50 mg in film-coated tablets.

The mean plasma concentration–time curves of metformin and vildagliptin obtained after a single oral administration of the test and reference products under fed conditions are shown in Figs. 1 and 2, which show superposable curves for both products.

Metformin HCl plasma concentrations were undetectable at predosing (00:00) for both the test and reference products.

The arithmetic mean C_max_ of metformin was 1693.76 ± 222.44 ng/mL for the test group and 1737.51 ± 211.99 ng/mL for the reference group.

The arithmetic means of metformin AUC_0→24 h_ and AUC_0→∞_ were 12798.6 ± 2268.79 ng·hr/mL and 13026.80 ± 2329.01 ng hr/mL, respectively, for the test product and 13002.57 ± 1722.89 ng hr/mL and 13263.02 ± 1782.65 ng·hr/mL, respectively, for the reference product.

Vildagliptin plasma concentrations were undetectable at predosing (00:00) and 24 h postdosing for both the test and reference products.

The arithmetic mean C_max_ of vildagliptin was 243.72 ± 70.11 ng/mL for the test group and 237.03 ± 55.20 ng/mL for the reference group.

The AUC_0→24 h_ and AUC_₀→∞_ arithmetic means for vildagliptin were identical and were 962.71 ± 199.45 ng hr/mL for the test product and 982 ± 2214.51 ng hr/mL for the reference product.

The major PK parameters of metformin and vildagliptin are summarized in Table 1.

**Table 1:** Main Pharmacokinetic parameters of Metformin and vildagliptin in both test and reference products.

Parameters	Metformin		Vildagliptin	
	Test (*N* = 18)	Reference (*N* = 18)	Test (*N* = 18)	Reference (*N* = 18)
	Arithmetic mean ± SD	Arithmetic mean ± SD	Arithmetic mean ± SD	Arithmetic mean ± SD
C_max_(ng/ml)	1693.76 ± 222.44	1737.51 ± 211.99	243.72 ± 70.11	237.03 ± 55.20
AUC_0→24 h_ (ng.hr/mL)	12798.60 ± 2268.79	13002.57 ± 1722.89	962.71 ± 199.45	982.00 ± 214.51
AUC_0→∞_ (ng.hr/mL)	13026.80 ± 2329.01	13263.02 ± 1782.65	962.71 ± 199.45	982.00 ± 214.51
T_max_ (hr)	3.75 ± 0.97	3.81 ± 0.97	1.99 ± 0.84	2.09 ± 0.99
T_1/2_ (hr)	4.06 ± 0.65	4.21 ± 0.57	1.94 ± 0.31	2.02 ± 0.39
Residual area (%)	1.71 ± 0.84	1.93 ± 0.87	0	0
λz (hr^−1^)	0.18 ± 0.04	0.17 ± 0.02	0.37 ± 0.06	0.35 ± 0.07

SD: standard deviation, λz: elimination rate constant, T_1/2_: terminal elimination half-life, T_max_: maximum concentration time, AUC_0→24 h_: area under the curve from zero to 24 h, AUC_0→∞_: area under the curve from zero to infinity, C_max_: maximum concentration, hr: *hour*, PK: pharmacokinetic.

The geometric mean T/R ratios (GMRs) of metformin for AUC_0→∞_, AUC_0→24 h_ and C_max_ were 97.45%, 98.68% and 97.33%, respectively. The geometric mean T/R ratios of vildagliptin for AUC_0→∞_, AUC_0→24 h_ and C_max_ were 98.19%, 98.19% and 101.56%, respectively. For bioequivalence evaluation, the PK parameters for both metformin and vildagliptin between the test and reference products, GMR, and 90% CIs were all within the range of 80–125%. See Table 2.

**Table 2:** Bioequivalence analysis of Metformin and vildagliptin after single oral administration of test and reference products.

Parameters	Geometric means		GMR%	90% CI	CV%
	Test (N = 18)	Reference (N = 18)			
Metformin					
C_max_(ng/ml)	1679.60	1725.66	97.33	92.01–102.66	14.11
AUC_0→24h_ (ng.hr/mL)	12596.34	12895.93	97.68	93.55–101.80	10.89
AUC_0→∞_ (ng.hr/mL)	12815.62	13150.77	97.45	93.32–101.58	10.93
Vildagliptin					
C_max_(ng/ml)	234.58	230.98	101.56	96.03–107.09	14.05
AUC_0→24h_ (ng.hr/mL)	945.25	962.70	98.19	94.46–101.92	9.80
AUC_0→∞_ (ng.hr/mL)	945.25	962.70	98.19	94.46–101.92	9.80

CI: confidence interval, AUC_0→24 h_: area under the curve zero to 24 h, AUC_0→∞_: area under the curve zero to infinity, C_max_: maximum concentration, GMR: geometric mean T/R ratio, CV%: intra-subject coefficient of variation.

### Safety assessment

Four subjects (two in each period) presented glucose levels below 70 mg/dL. Overall, 6 out of 108 (5.5%) blood glucose measurements were below 70 mg/dL. The assessment of other vital signs, including blood pressure, heart rate, and temperature, did not reveal any clinically significant changes in either period. No serious adverse events or unexpected adverse events occurred during this study for the test or reference products.

Subject 1 was given 60 mL of 20% glucose solution orally twice and kept under monitoring until his blood glucose was normal, as described in the study protocol.

All observed AEs recovered within a few hours and did not require any further investigations.

## Discussion

To the best of our knowledge, this is the first bioequivalence study to assess the bioequivalence of two formulations of metformin/vildagliptin in healthy Tunisian volunteers under fed conditions. Our study demonstrated that the new generic film-coated tablet combination of metformin hydrochloride/vildagliptin (850 mg/50 mg) was bioequivalent to the same film-coated tablet strength of the marketed reference. These results support the marketing of this new generic in Tunisia.

Intrasubject variability of C_max_ is crucial in bioequivalence trials, as it is a pivotal parameter for sample size determination and thereby influences overall trial validity^12^. Unfortunately, these valuable data are often limited in the literature, and in their absence, intersubject variability is frequently utilized, potentially leading to an overestimation of the required sample size^13–15^. In our investigation, we determined the intrasubject variability for the C_max_ of metformin and vildagliptin to be 14.11% and 14.05%, respectively. Notably, these findings align with literature, where the reported intrasubject variability for vildagliptin C_max_ was 15.85%, demonstrating consistency^8^. However, our observed intrasubject variability for metformin C_max_, at 14.11%, diverges from the literature-reported value of 8.95% ^8^. These results emphasize the importance of considering intrasubject variability in bioequivalence trials and underscore the need for accurate and context-specific data to inform sample size calculations. The nuanced variability between our study and the literature values for metformin further underscores the importance of discerning drug-specific characteristics when designing and interpreting bioequivalence studies.

Our sample size of 18 subjects provided a statistical power of 90% to demonstrate bioequivalence, consistent with regulatory expectations. Previous bioequivalence studies on metformin/vildagliptin have reported variable sample sizes, ranging from 12 to 60 participants. For example, Schnaars et al. conducted a study with a cohort of 60 subjects, with 90% power but different intrasubject variability^16^. Another bioequivalence study involving only 12 subjects reported the equivalence of the two drug formulations^8^. While many regulatory agencies require a minimum of 12 subjects^5,12^, relying on such a small cohort may compromise study validity due to the inherent risk of withdrawals or protocol deviations. Furthermore, conducting a bioavailability study with more than 40 subjects is generally deemed impractical^13^. This underscores the delicate balance that researchers must strike in determining an appropriate and feasible sample size for bioequivalence trials. Ethical considerations also play a role in determining subject numbers to avoid unnecessary exposure of healthy volunteers. In such cases, accepting lower statistical power may become unavoidable. However, a larger subject cohort beyond the defined statistical power (80%) may narrow confidence intervals. In studies involving healthy volunteers in particular, financial and ethical constraints can pose challenges^13^.

The selection of fed conditions for this trial was driven by the pharmacokinetic characteristics of the two drugs. Data from the literature have shown that both vildagliptin and metformin pharmacokinetics are not significantly influenced by age, sex, or BMI^16^; therefore, the inclusion criteria for the present study were set as aforementioned without broad restrictions.

Vildagliptin exposure is minimally affected by food, although food intake may delay T_max_. In contrast, metformin absorption is known to be significantly influenced by food, with reduced Cmax but generally unchanged total exposure. Therefore, a fixed-dose combination of vildagliptin and metformin bioavailability was assessed in this study under fed conditions through a two-way crossover design^2,16^.

In their open-label study under both fed and fasting conditions, He et al. demonstrated that while food did not impact the pharmacokinetics of vildagliptin, it slowed the absorption rate of metformin without altering its overall extent of absorption. As a result, the fixed-dose combination tablet of vildagliptin and metformin can be administered according to the same guidelines as metformin regarding food intake^17^.

Overall, the pharmacokinetic findings of our study are consistent with previously published data and confirm that the test FDC formulation provides comparable systemic exposure to the reference product. The demonstration of bioequivalence under fed conditions supports the clinical substitutability of the test product and contributes to the availability of a locally manufactured alternative for the management of type 2 diabetes in Tunisia. Demonstrating bioequivalence is a critical regulatory requirement not only for initial marketing authorization but also for ensuring interchangeability with existing therapies. Furthermore, establishing BE supports broader public-health objectives by enabling access to cost-effective therapies and improving adherence.

## Conclusion

In this single-dose crossover clinical trial conducted under fed conditions, the test product Bi-Galvine 850/50 mg Film-Coated Tablets of SAIPH Pharma, Tunisia, showed bioequivalence to the reference product Galvumet 850/50 mg Film-Coated Tablets of Novartis Pharma Schweiz AG, Risch, Suisse, in terms of the rate and extent of absorption.

## Data Availability

Prof. Sameh Trabelsi M.D, head of the Clinical Pharmacology Department could be contacted if data from this study are requested.
